# Genetic and environmental control of the *Verticillium *syndrome in *Arabidopsis thaliana*

**DOI:** 10.1186/1471-2229-10-235

**Published:** 2010-11-02

**Authors:** Eva Häffner, Petr Karlovsky, Elke Diederichsen

**Affiliations:** 1 Freie Universität Berlin, Institut für Biologie - Angewandte Genetik, Albrecht-Thaer-Weg 6, 14195 Berlin, Germany; 2 Georg-August-Universität Göttingen, Department of Crop Science, Molecular Phytopathology and Mycotoxin Research Unit, Grisebachstraße 6, 37077 Göttingen, Germany

## Abstract

**Background:**

*Verticillium *spp. are major pathogens of dicotyledonous plants such as cotton, tomato, olive or oilseed rape. *Verticillium *symptoms are often ambiguous and influenced by development and environment. The aim of the present study was to define disease and resistance traits of the complex *Verticillium longisporum *syndrome in *Arabidopsis thaliana *(L.) Heynh. A genetic approach was used to determine genetic, developmental and environmental factors controlling specific disease and resistance traits and to study their interrelations.

**Results:**

A segregating F2/F3 population originating from ecotypes 'Burren' (Bur) and 'Landsberg *erecta*' (L*er*) was established. Plants were root-dip inoculated and tested under greenhouse conditions. The *Verticillium *syndrome was dissected into components like systemic spread, stunting, development time and axillary branching. Systemic spread of *V. longisporum *via colonisation of the shoot was extensive in L*er*; Bur showed a high degree of resistance against systemic spread. Fungal colonisation of the shoot apex was determined by (a) determining the percentage of plants from which the fungus could be re-isolated and (b) measuring fungal DNA content with quantitative real-time PCR (qPCR). Four quantitative trait loci (QTL) controlling systemic spread were identified for the percentage of plants showing fungal outgrowth, two of these QTL were confirmed with qPCR data. The degree of colonisation by *V. longisporum *was negatively correlated with development time. QTL controlling development time showed some overlap with QTL for resistance to systemic spread. Stunting depended on host genotype, development time and seasonal effects. Five QTL controlling this trait were identified which did not co-localize with QTL controlling systemic spread. *V. longisporum *induced increased axillary branching in Bur; two QTL controlling this reaction were found.

**Conclusions:**

Systemic spread of *V. longisporum *in the host as well as resistance to this major disease trait are described for the first time in natural *A. thaliana *accessions. This creates the possibility to study a major resistance mechanism against vascular pathogens in this model plant and to clone relevant genes of the involved pathways. Stunting resistance and resistance to systemic spread were controlled by different QTL and should be treated as separate traits. Developmental and environmental effects on pathogenesis and resistance need to be considered when designing and interpreting experiments in research and breeding.

## Background

*Verticillium *spp. are vascular fungal pathogens that induce diverse disease symptoms and severe yield losses on a broad range of dicotyledonous plants [1]. Disease symptoms include wilting, chlorosis, stunting, vascular discoloration, defoliation or premature seed ripening. Infected plants often show unspecific reactions similar to those which occur during senescence or under environmental stress conditions.

In susceptible crops the infection starts in the root and proceeds into the shoot by systemic spread via the transpiration stream in the xylem [2,3]. Certain conditions, for example developmental events, can influence the initiation of the systemic phase [3]. Resistance to systemic spread has been shown to be a major component of *Verticillium *resistance for many hosts [3-9]. Resistance to systemic spread is regarded as a type of resistance that reduces the rate of epidemic development in the field [10]. Rate-reducing resistance has been shown to be a major component of field resistance in economically important crops. In soybean for example, yield depends strongly on rate-reducing resistance against Phytophthora root and stem rot [11] and sudden death syndrome caused by *Fusarium *[12]. In both cases a major component of rate-reducing resistance is the host's ability to restrict fungal colonisation of the plant tissue [12,13].

*V. longisporum *(Stark) Karapapa is a soil-borne fungal pathogen specialised for cruciferous hosts [14]. During the last decades, it has become a serious threat to oilseed rape in Central and Northern Europe [15,16]. Since there are no efficient fungicides available against this pathogen, resistance breeding is the most promising approach to control the disease. To date, only quantitative resistance against *V. longisporum *is known [17]. Quantitative resistance is characterised by being incomplete and conditioned by multiple genes of partial effect [18]. Often, quantitative resistance to pathogens is a pleiotropic effect of genes affecting growth and development [18,19]. The situation in the *V*. *longisporum-*Brassicaceae-pathosystem is further complicated by the diversity of the symptoms induced. Chlorosis and stunting are among the most obvious symptoms in root-dip inoculated *Brassica *plants and are often used to assess disease progression in greenhouse experiments [4,20,21]. Mapping of resistance QTL has mostly been performed on the basis of these symptoms [22]. However, stunting has never been observed in the field. The prevalent symptom on oilseed rape in the field is premature ripening [23], which is accompanied by systemic spread, extensive formation of microsclerotia on shoot tissue, and yield loss [24]. Systemic spread in the host plant has been shown to be a specific component of the *V. longisporum-Brassica*-interaction. Whereas *V. dahliae*-infection is mainly restricted to the roots, *V. longisporum *is capable of invading the shoot system of susceptible *Brassica *genotypes [2,3]. The transition to flowering has been shown to be a crucial developmental stage promoting systemic spread [3], but in extremely susceptible genotypes systemic spread has been observed in even earlier stages [24]. Symptom severity corresponded with the presence of *V. longisporum *in the shoot system for *Brassica *spp. in greenhouse experiments [4,5]. Systemic spread is regarded as a main indicator for disease severity in the field [24]. The genetic basis of resistance to systemic spread in *Brassica *remains unknown.

Recent studies used *A. thaliana *as a model organism to further elucidate the genetic basis of *Verticillium *resistance and disease traits [25-29]. *V. dahliae *and *V. longisporum *are capable of inducing symptoms in *A. thaliana *comparable to those in *Brassica*. Differences in symptom severity have been observed among natural accessions of *A. thaliana *or in specified mutants. A locus on chromosome 4, *Vet1*, was shown to confer resistance to chlorosis and it also delayed flowering after *Verticillium *infection [29]. Two QTL were mapped to different chromosomal positions that were associated with resistance to chlorosis, and a new allele of *rfo1 *(resistance to *Fusarium oxysporum*) was described that increased the resistance to fresh weight loss caused by *V. longisporum *[26]. By testing defined *A. thaliana *mutants for their reaction towards *Verticillium *infection, it has been shown that processes as diverse as ethylene signalling [26,27,29], R-gene signalling [26] and post-transcriptional gene-silencing [25] are involved in resistance against *Verticillium*. Disease severity was recorded as stunting, fresh weight loss and chlorosis. In two of these studies the degree of colonisation by *V. longisporum *in the host was recorded. The higher susceptibility of ecotype 'Col-0' to *Verticillium*-induced chlorosis was not accompanied by higher numbers of colony-forming units in whole plants when compared to ecotype 'C24' [29]. Mutants with impaired endogenous gene silencing showed increased *Verticillium *susceptibility in terms of stunting and chlorosis and also more fungal biomass compared to wild type [25]. Relatively few research studies focus on the natural genetic resources for *V. longisporum*-resistance. *Brassica *as well as *A. thaliana *accessions may still harbour numerous unknown genes capable of influencing the interaction with *V. longisporum *[17,20,26].

The aim of the present study was to localise genomic regions in *A. thaliana *that influence disease and resistance traits in order to identify the associated genes and pathways using a QTL mapping approach. An F2/F3 mapping population was derived from a cross between two ecotypes that displayed striking differences in their interaction with *V. longisporum*. Special emphasis was placed on the definition of disease and resistance traits such as systemic spread and stunting. The degree of systemic fungal spread into the apical parts of the shoot, a trait whose natural variation has not been examined in a genetic study of *A. thaliana *before, was determined with two different methods: Re-isolation from apical shoot segments and qPCR of fungal DNA. To analyse the developmental implications of the disease the branching pattern and the duration of the development were recorded. To study disease traits in closer similarity to the natural situation, a greenhouse testing procedure was used. The dissection of the complex syndrome in a genetic study allowed the detection of QTL controlling different traits, to investigate their relationships and to draw conclusions regarding their role for disease and resistance.

## Methods

### Material

*A. thaliana *ecotypes Bur-0 and L*er*-0 were originally obtained from the Arabidopsis Information Service (AIS) Frankfurt [30] and maintained in house. The *V. longisporum *isolate '43' [31] was used for infestation experiments.

### Fungal culture and preparation of spore suspension

Fungal stocks with 1-3×10^6 ^conidia/ml were stored in glycerol:water (1:4) at -75°C. Conidial suspensions for inoculation were produced in liquid Czapek-Dox medium on a shaker at 20°C for 8 days and filtered through sterile gauze. Spore densities were determined using a haemocytometer (Neubauer improved). For inoculation, spore suspensions were diluted to 1×10^6 ^conidia/ml with sterile tap water.

### Infestation experiments

For all greenhouse infestation experiments, a root-dip inoculation procedure [31] was applied which was modified for *A. thaliana *as follows. Seeds were stratified at 8°C for 2 days before sowing and plantlets were grown in a mix of commercial potting soil (Einheitserde Typ P) and sand (3:1 vol. parts) under long-day conditions (16 h light) at 20°C for 19 days. Supplementary lighting was provided using sodium vapour lamps. After uprooting and cutting the root tips plantlets were dipped in a conidial suspension for 1 h. Controls were mock-inoculated in diluted Czapek-Dox medium without spores. Thirty plants were planted per 20×30 cm plastic tray filled with 1.5 l soil/sand mix. Inoculated plants were grown in long-day in the greenhouse at 18-28°C until maturity of the first siliques. Then the height of the aerial parts of the plant (from the hypocotyl to the tip of the longest stalk), their fresh weight, the branching pattern and colonisation of the apical part of the shoot were determined. To test whether infection requires root injury, individual plantlets were raised in pots of 5 cm diameter for 19 days as described above, and then 5 ml of conidial suspension were pipetted into the soil next to the hypocotyls of the seedling. Three F3 infestation experiments were carried out. Experiment 1 (E1) was started in January (winter experiment), experiment 2 (E2) in March and experiment 3 (E3) in April (referred to as spring experiments). Altogether, 108 families were tested. Each experiment comprised 60 F3-families which were partly overlapping between the experiments. The overlap was ten F3-families between E1 and E2, 31 between E2 and E3, and 32 between E1 and E3. Ten selected F3-families, parents and F1 were analysed in every experiment. Of each F3-family, 30 inoculated and 15 mock-inoculated plants were tested. The parental ecotypes L*er *and Bur were tested at least eleven times during all seasons of the year with at least 30 replicates per experiment.

### Determination of systemic fungal spread

Systemic spread of *V. longisporum *was observed to occur during flowering (see Results) and was therefore determined at the onset of silique maturity. To determine systemic spread by re-isolation, segments of approx. 3 cm length were cut from the apex of the main shoot axis of each plant, surface-sterilised with 0.1% (w/v) sodium hypochlorite solution and subsequently dipped in 70% (v/v) ethanol. After rinsing three times in sterile water, the segments were transferred to Petri dishes containing 9 g/l agar, 10 g/l malt extract and 100 mg/l streptomycin. *V. longisporum *outgrowth was recorded after 1 and 2 weeks. One shoot segment per plant was plated and the percentage of colonised shoot segments of the total number of shoot segments per F3-family was calculated (degree of colonisation). In experiment E3, apical pieces of the main shoot of parents, F1 and F3-families were also analysed for the amount of fungal DNA in the plant tissue by quantitative real-time PCR. Standards of *V. longisporum *DNA were prepared as described [32]. Real-time PCR based on internal transcribed sequences of ribosomal RNA genes with SYBR Green fluorescence monitoring was used for the quantification of fungal DNA in plant tissue [2]. The primers were designed to amplify *Verticillium *spp. but not other organisms potentially infecting *A. thaliana*. Usually three samples consisting of a mixture of approx. 10 plants were used per family.

### Assessment of the developmental stage

The following scale was applied: Stage 0 = vegetative; stage 1 = first buds visible; stage 2 = first bud > 1 mm, flowering shoot elongating; stage 3 = 1-3 flowers open; stage 4 = 4-10 flowers open; stage 5 = more than 10 flowers open; stage 6 = 1-3 siliques mature; stage 7 = 4-6 siliques mature; stage 8 = up to 50% of the siliques on main shoot mature; stage 9 = more than 50% of siliques on main shoot mature; stage 10 = all siliques mature, whole plant yellow.

### Generation of the mapping population

A Bur-0 ♀ × L*er*-0 ♂ cross was performed. A single F1-plant was selfed and 243 F2-plants were raised from which leaf material for marker analysis was collected. Each F2-plant was selfed again and F3-seeds were collected from each F2-plant separately. Parents, F1 and F3-families were used for infestation experiments.

### Marker analysis

The markers used in the present study were identified from different sources. Twenty eight previously published SSR markers [33,34] were used. Eleven sequence-characterised (SCAR) markers were developed for the present study (see Additional File 1) by exploiting length polymorphisms in the sequences of L*er*-1 and Bur-0 [35] using the MSQT query tool [36]. The *erecta *mutation present in L*er *[37] was used as a morphological marker. DNA of shock-frozen rosette leaves was extracted with the CTAB method [38]. The markers were amplified using PCR (94°C for 1 min, 40 cycles of 94°C for 15 s, 50 or 55°C for 15 s, 72°C for 30 s). PCR products were separated on 3.5-4% (w/v) agarose gels (Agarose NEEO Ultra from Roth).

### QTL analysis

A linkage map of 40 markers polymorphic for the parental lines was constructed and allele frequencies of each marker were analysed for significant deviations from the 1:2:1 segregation ratio with the program MapDisto [39]. QTL analysis was performed with MapManager QTX 20b [40] using the Kosambi function. Simple interval mapping was performed scanning the genome in 1 cM-steps. In cases where one locus was especially prominent, composite interval mapping using the most significant locus as a background for the calculation of further QTL was performed. MapManager QTX gives the LRS (likelihood ratio statistic) value to assess the probability of a false positive, where LRS = 4.6 × LOD [41]. LRS significance threshold values for the 37% (putative), 95% (significant) and 99.9% (highly significant) genome-wide confidence level were determined by permutation tests with 10,000 permutations. The position of a QTL was determined as a peak LRS score exceeding a certain confidence level. Confidence intervals for QTL were determined by bootstrap tests which calculate the QTL position for multiple resampled data sets of the original data set. Only those QTL were considered which were detected either in all three F3 experiments or were detected in at least two experiments and beyond the genome-wide 95%-significance threshold in at least one experiment. For the detection of epistatic interactions the confidence criterion of p = 10^-5 ^was set.

### Statistical analyses

Frequency distributions of trait values for all F3-families within an infestation experiment were tested for normality with the Kolmogorow-Smirnow-test and the Shapiro-Wilk test. For quantitative assessment of the degree of axillary branching, a median test was performed: the number of plants having an axillary branching score above the overall median was recorded for each F3-family, F1 and the parental lines. In order to determine the origin of the increasing allele and to estimate the inheritance pattern of a certain QTL, ANOVA and multiple comparisons (Tukey test) were performed on trait values between families which were maternal, paternal or heterozygous at the marker site closest to the QTL of interest. For those traits which did not show a normal distribution, the results were confirmed with the non-parametric Mann-Whitney-U-test. The U-test was also used to compare the values from the median test quantifying basal axillary branching. Heritability for traits which could be measured for single plants was calculated as H^2 ^= V_G_/V_G_+V_E _using variance components obtained through MINQUE, where V_G _is the genetic variance and V_E _is the environmental variance [42]. For traits with single values per F3-family, like the degree of systemic colonisation or axillary branching scores, heritability was calculated after the F3P method [43] using the formula H^2 ^= V_F3_-_P_/V_F3, _where V_F3 _is the variance between the F3-families and _P _is the average variance of a trait within the parental lines. To test for genotype × experiment-interactions, two-factorial ANOVA was performed for all traits with single-plant values. SPSS Statistics 17.0 (SPSS Inc., Chicago) was used for all statistical analyses.

## Results

### Establishment of a linkage map for (Bur×L*er*) mapping populations

The *A. thaliana *ecotypes Landsberg *erecta *(L*er*) and Burren (Bur) were found to differ in their reactions towards challenge with the vascular pathogen *V. longisporum *in various aspects (see below). To identify chromosomal regions that control the traits, a (Bur×L*er*) map based on 28 simple sequence repeat (SSR) markers, 11 sequence-characterised (SCAR) markers and one morphological marker (*erecta*) was established. The marker locations on the physical map of the Arabidopsis Genome Iniative (AGI) are known for most markers and the marker order of the linkage map was as predicted according to the physical positions. The SCAR markers were developed for the present mapping population from known insertions or deletions in the Bur and L*er *genomes and are presented in Additional File 1. In total the map spanned 368.2 cM, with an average marker spacing of 9.4 cM (see Additional File 2). The largest distance between adjacent markers was 24.4 cM. Segregation distortion was only observed at marker EH3-1 on chromosome 3.

### Multiple QTL control resistance to systemic spread in *A. thaliana *ecotype Bur

**Figure 1:**
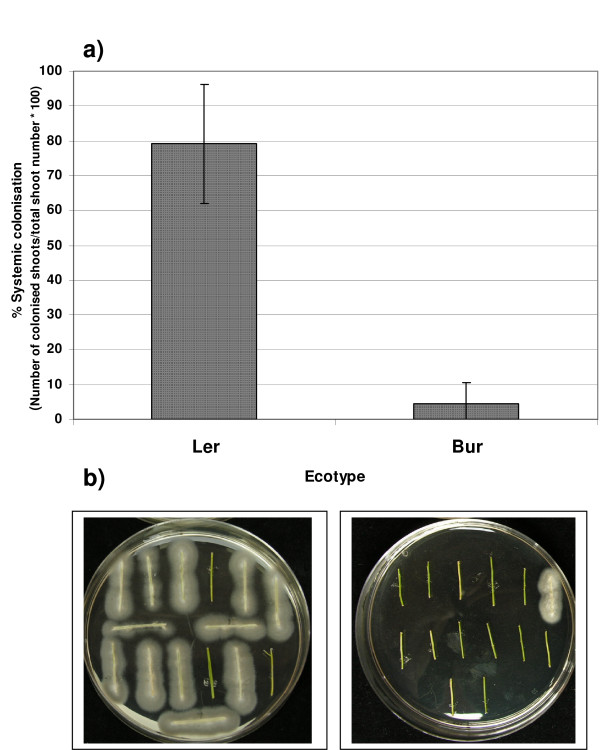
**Degree of systemic colonisation by *V. longisporum *in L*er *and Bur at the onset of maturity**. a) Percentage of plants with colonised apical shoot segments (100% = total number of tested plants). Means of nine experiments are shown, bars denote standard deviations. b) L*er *shoot segments (left) showing heavy outgrowth of *V. longisporum*, sparse outgrowth from Bur shoot segments (right).

**Figure 2:**
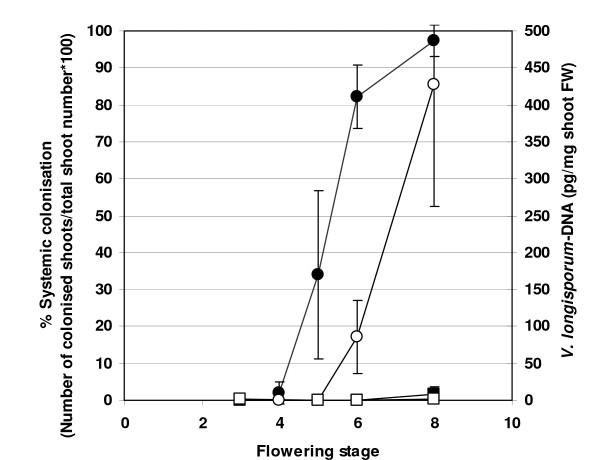
**Systemic colonisation of L*er *and Bur by *V. longisporum *in the course of plant development**. Percentage of colonised plants (closed symbols) and fungal DNA-content in apical shoot segments (open symbols) in L*er *(circles) and Bur (squares) at different developmental stages. Developmental stages: 3 = 1-3 flowers open, 4 = 4-10 flowers open, 5 = more than 10 flowers open, 6 = up to 3 siliques mature, 7 = 4-6 siliques mature, 8 = up to 50% of siliques on main shoot mature. N = 4 (Bur) or 5 (L*er*), bars denote standard deviations.

**Figure 3:**
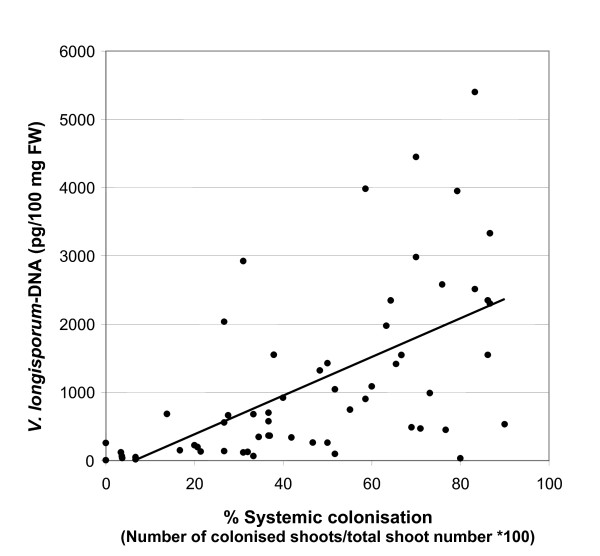
**Correlation of data from two methods for quantifying systemic spread of *V. longisporum***. Correlation between the percentage of colonised plants (malt agar test) and the mean *V. longisporum *DNA content in F3-families tested in experiment E3 (r = 0.59, p < 0.001).

Significant differences in the extent of systemic spread of *V. longisporum *in the shoot system were found between the *A. thaliana *ecotypes L*er *and Bur in greenhouse infestation experiments. The degree of systemic colonisation was measured as the percentage of apical shoot segments colonised by the fungus compared to the total number of shoot segments. When explanted on malt agar plates, apical shoot segments of inoculated L*er *plants showed fungal outgrowth to a much higher percentage than those of ecotype Bur at the onset of fruit maturation (Figure 1), reflecting a difference in the probability of viable conidiospores reaching the apical part of the shoot system. To study the progress of infection during plant development, the time course of systemic spread was analysed for both ecotypes. In addition, the amount of fungal DNA in apical shoot segments was measured by qPCR. In L*er*, shoot colonisation started during the early flowering stages and reached highest values during fruit maturation, whereas the colonisation levels in Bur were low throughout all developmental stages (Figure 2). The onset of fruit maturation was chosen as the most appropriate time point to determine the degree of systemic colonisation in all subsequent infestation experiments because by then colonisation was already advanced in susceptible lines and the shoot tissue was still viable enough so that no saprophytic growth could take place. Since Bur develops more slowly than L*er*, and in routine experiments both ecotypes were inoculated at the same time, the possibility had to be considered that the time point of inoculation relative to the onset of flowering was responsible for the difference in systemic spread. To test this hypothesis, both ecotypes were inoculated at specific developmental stages. L*er *always showed high colonisation rates, whereas Bur always showed low colonisation rates (data not shown). Thus the difference in systemic spread between the ecotypes was not caused by differences in the developmental stage during inoculation. It was further tested whether the difference in systemic spread could also be observed when plants were inoculated without prior wounding of the roots. The colonisation rates in this experiment were 0% for Bur and 90% for L*er *and thus very similar to the rates in experiments with dip inoculation of injured roots. For QTL-mapping of loci affecting systemic spread, the percentage of plants showing colonised shoot segments was recorded in F3-families originating from individual F2-plants in three infestation experiments (E1-E3). For E3, data from qPCR quantifying the amount of *Verticillium*-DNA in the shoot samples were also available. Means of *V. longisporum-*DNA amounts determined by qPCR were significantly correlated with the colonisation values of the malt agar test in E3 (Figure 3). Broad-sense heritability (H^2^) of the trait systemic colonisation was moderate to high when data of the malt agar test were used. With the qPCR values available for E3, H^2 ^was much lower (Table 1). Frequency distributions of colonisation data in each experiment were multi-modal and suggest that several QTL are involved in controlling this trait (Additional File 3).

**Table 1:** Broad-sense heritability for *Verticillium*-related traits in the *A. thaliana *(Bur×L*er*) F2/F3 mapping population.

Trait	**Exp**.	**H**^**2**^	Method
Degree of *Verticillium *colonisation (*vec*; malt agar test; number of colonised shoot segments/total number of shoot segments in%)	E1	0.58	F3P
	E2	0.59	
	E3	0.76	

Degree of *Verticillium *colonisation (*vec a*; real-time qPCR; pg *Verticillium *DNA/100 mg shoot fresh weight)	E3	0.45	VCA

Development time (*dt*; days from germination to maturity in mock-inoculated plants)	E1	0.46	VCA
	E2	0.49	
	E3	0.62	

Shoot fresh weight (g)	E1	0.21	VCA
	E2	0.40	
	E3	0.49	

Stunting resistance (*stre*; shoot height of *Verticillium*-inoculated plants in cm)	E1	0.35	VCA
	E2	0.49	
	E3	0.39	

*Verticillium*-induced axillary branching (*vab*; number of plants with a branching score above the overall median of *Verticillium*-inoculated plants)	E1	0.54	F3P
	E2	0.67	
	E3	0.82	

Broad-sense heritability (H^2^) of traits for each experiment (exp.) was calculated either with variance component analysis (VCA) [42] or with the F3P method [43] as indicated.

**Figure 4:**
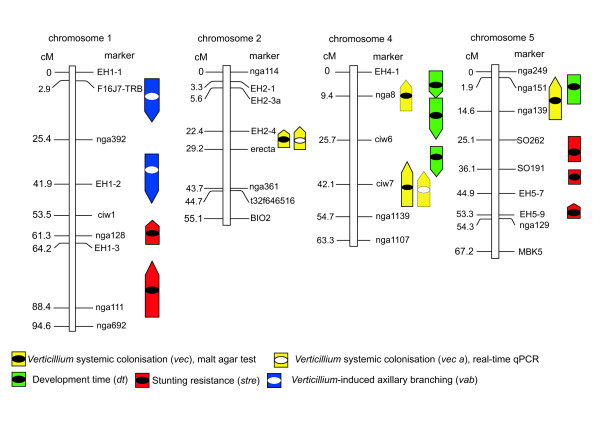
**Localisation of QTL controlling *Verticillium *resistance and disease-related traits in *A. thaliana***. The length of the bars denotes confidence intervals as determined by bootstrap tests, ellipses indicate the peak positions of the LRS. Upward arrows indicate the paternal parent (L*er*) as the source of the increasing allele, downward arrows the maternal parent (Bur). Bars without arrows stand for QTL for which the parental effect was ambiguous. Dotted bars stand for QTL with LRS values below the genome-wide 95% significance level which were included for their reproducibility (see text, Table 2 and Additional File 4). Results are displayed for the most representative experiment. For further information on QTL see Table 2 and Additional File 4.

Four QTL that meet the criteria of significance and/or reproducibility applied in the present study were detected by interval mapping of the trait systemic colonisation as determined by the malt agar test (Figure 4, Table 2). The QTL with the strongest effect (*vec1*) was detected on chromosome 2 near the morphological marker *erecta*. Three QTL of lesser effect (*vec2*, *vec3 *and *vec4*) were detected near the markers nga8 and ciw7 on chromosome 4 and near marker nga139 on chromosome 5, respectively. *vec2 *and *vec4 *were detected in all three infestation experiments, whereas *vec1 *and *vec3 *could be reproduced in two of three experiments. Results from the most representative experiment for each QTL were chosen (see also Additional File 4) and displayed in Figure 4 and Table 2. All *vec *alleles increasing the degree of colonisation came from ecotype L*er*. *vec1*, *vec2 *and *vec3 *were recessive, *vec4 *showed intermediate inheritance (Table 2). This corresponds with low to moderate colonisation rates in the F1-generation (see Additional File 3). When QTL-mapping was performed with fungal DNA contents in experiment E3, two QTL could be detected whose confidence intervals overlapped with *vec1 *and *vec3*, respectively (Figure 4, Table 2). The peak LRS values of *vec1 *and *vec3*, however, were lower when qPCR data were applied (Table 2, Additional file 4). *vec2 *and *vec4*, which were only detected at the putative level with the re-isolation method in E3, could not be detected with the qPCR values. Epistatic interactions were analysed with MapManager QTX. One epistatic interaction was detected in experiment E3 between marker *erecta*, which is close to *vec1*, and marker ciw6 on chromosome 4, which is not within the confidence interval of any of the *vec *QTL reported here. This interaction was not detected in E1 or E2 or by qPCR.

**Table 2:** QTL detected for *Verticillium*-related traits in the *A. thaliana *(Bur×L*er*) F2/F3 mapping population.

Trait	QTL	**Exp**.	**Chr**.	Peak pos. (cM)	Nearest marker	LRS	LOD	Explained trait variance	Mean ± SD (or median) for F3-families with alleles from:		
									Bur	Heterozygous	L*er*
Degree of *Verticillium *colonisation (*vec*; malt agar test; number of colonised shoot segments/total number of shoot segments in%)	*vec1*	E1	2	26	erecta	21.2**	4.6	31%	20.4 ± 13.8^a^	18.4 ± 14.5^a^	43.8 ± 27.1^b^
	*vec2*	E2	4	10	nga8	9.4*	2.0	11%	21.2 ± 17.2^a^	26.3 ± 21.8^a^	41.8 ± 15.8^b^
	*vec3*	E3	4	45	ciw7	17.0**	3.7	18%	32.0 ± 29.2^a^	41.0 ± 20.5^a^	65.2 ± 21.5^b^
	*vec4*	E1	5	11	nga139	20.7**	4.5	31%	7.7 ± 6.0^a^	23.2 ± 16.5^b^	42.0 ± 24.1^c^

Degree of *Verticillium *colonisation (*vec a*; real-time qPCR; pg *Verticillium *DNA/100 mg shoot fresh weight)	*vec1 a*	E3	2	27	erecta	14.2**	3.1	20%	764 ± 1071^a^	1061 ± 1035^a^	1994 ± 1588^b^
	*vec3 a*	E3	4	47	ciw7	13.4*	2.9	13%	976 ± 1162^a^	751 ± 741^a^	2015 ± 1615^b^

Development time (*dt*; days from germination to maturity in mock-inoculated plants)	*dt1*	E1	4	5	nga8	18.8**	4.1	29%	64.4 ± 7.5^a^	58.6 ± 7.4^a,b^	54.3 ± 3.8^b^
	*dt2*	E1	4	16	nga8	18.2**	4.0	28%	64.4 ± 7.5^a^	58.6 ± 7.4^a,b^	54.3 ± 3.8^b^
	*dt3*	E1	4	33	ciw6	17.2**	3.7	26%	63.3 ± 7.2^a^	58.0 ± 7.2^a,b^	54.5 ± 4.7^b^
	*dt4*	E2	5	5	nga151	17.0**	3.7	24%	50.3 ± 3.1^a^	55.0 ± 4.8^b^	49.8 ± 5.7^a^

Stunting resistance (*stre*; shoot height of *Verticillium*-inoculated plants in cm)	*stre1*	E1	1	79	nga111	32.5***	7.1	51%	10.9 ± 2.2^a^	16.1 ± 3.8^b^	20.2 ± 4.3^c^
	*stre2*	E1	1	59	nga128	22.2**	4.8	39%	11.1 ± 2.3^a^	17.4 ± 4.5^b^	17.8 ± 3.9^b^
	*stre3*	E3	5	31	SO191	20.4**	4.4	39%	28.7 ± 3.1^a,b^	24.7 ± 5.3^a^	32.7 ± 3.5^b^
	*stre4*	E3	5	39	SO191	20.9**	4.5	40%	28.7 ± 3.1^a,b^	24.7 ± 5.3^a^	32.7 ± 3.5^b^
	*stre5*	E3	5	53	nga129	15.8**	3.4	32%	26.5 ± 3.1^a^	25.7 ± 5.5^a^	33.5 ± 3.2^b^

*Verticillium*-induced axillary branching (*vab*; number of plants with a branching score above the overall median of *Verticillium*-inoculated plants)	*vab1*	E1	1	9	F16J7-	28.1***	6.1	39%	20^a^	13^b^	10^b^
					TRB						
	*vab2*	E1	1	34	EH1-2	22.2**	4.8	33%	20^a^	12^a,b^	10.5^b^

LRS = likelihood ratio statistic. Asterisks denote genome-wide significance thresholds determined by permutation tests as follows: * 37%, ** 95%, ***99.9%. In each row, values with different letters differ significantly with at least p < 0.05. ANOVA and tukey tests were used for metric values; differences of values that were not normally distributed were confirmed with pairwise Mann-Whitney-U-tests. For axillary branching scores the median is given; values were compared pairwise with U-tests. Results are displayed for the most representative experiment (exp.). For reproducibility see Additional file 4.

### QTL controlling systemic spread partly overlap with QTL for development time

The degree of colonisation was negatively correlated with the duration of development among F3-families in all three experiments (Table 3). QTL for development time were therefore mapped by recording the number of days from germination to the maturation of the first siliques in mock-inoculated plants. Bur and L*er *differed in the duration of their developmental cycle, reflecting the difference in flowering time. The time until the plants reached maturity was always longer for Bur than for L*er*, but for both ecotypes it varied considerably with the season in which the experiment was performed. In summer experiments, the fastest development for L*er *was 32 days to maturity, for Bur 38 days. In winter, the longest period for both ecotypes was 60 and 72 days to maturity respectively. The segregating F3-population showed transgressive variation in one direction: No F3-family had a shorter mean development time than L*er*, but up to 40% of all F3-families and, in two experiments, also the F1-generation showed a slower development than Bur (Additional File 3). Family means of development time for the controls were normally distributed in all three experiments (Additional File 3). Broad sense heritability of the trait was moderate (Table 1). Development time was significantly affected by genotype and by the experiment, and significant interactions between genotype and experiment were found (Additional File 5). Three QTL controlling development time were found in the top half of chromosome 4 (*dt1-dt3*), and one QTL was detected near marker nga151 on chromosome 5 (*dt4*; Figure 4, Table 2). *dt1 *and *dt4 *were detected in every experiment, *dt2 *and *dt3 *only in two of three F3 experiments (Additional File 4). The QTL with the strongest effect, *dt1*, was mapped near marker nga8 at the top of chromosome 4 and thus co-localised with a major colonisation QTL, *vec2*. For the three *dt *QTL on chromosome 4, the alleles delaying development came from Bur, whereas for *dt4*, families originating from heterozygous F2-plants flowered significantly later than either maternal or paternal homozygous families. An epistatic interaction could be detected in E1 and E2 between marker EH4-1 on chromosome 4, which is in the confidence interval of *dt1*, and marker SO191 on chromosome 5. In E2, a second interaction was detected between EH4-1 and nga151, the latter being within the confidence interval of *dt4 *on chromosome 5.

**Table 3:** Correlations between resistance and developmental traits in F3-families.

Traits	**Exp**.	r
Degree of *Verticillium *colonisation **vs**. days to maturity in mock-inoculated plants	E1	-0.42**
	E2	-0.73***
	E3	-0.62***

*Verticillium*-induced difference in days to maturity **vs**. days to maturity in mock-inoculated plants	E1	0.74***
	E2	0.61***
	E3	0.68***

Performance 'Height' **vs**. days to maturity in mock-inoculated plants	E1	-0.67***
	E2	-0.43***
	E3	-0.47***

Performance 'Height' **vs**. degree of *Verticillium *colonisation	E1	0.47***
	E2	0.26*
	E3	0.27*

Exp. = experiment, r = Pearson correlation coefficient. Asterisks denote error probability (* α = 5%, ** α = 1%, *** α = 0.1%).

*V. longisporum*-treatment significantly accelerated the development in many F3-families. Acceleration (mean days to maturity_controls _- mean days to maturity_inoculated_) was closely correlated with the duration of the developmental cycle in controls (Table 3), i.e. slowly developing families were more accelerated after infection than fast families. The correlation was much weaker with the days to maturity of the inoculated variant (r = 0.39** in E1). QTL controlling developmental acceleration, however, could not be detected.

### Stunting depended on development time and season

**Figure 5:**
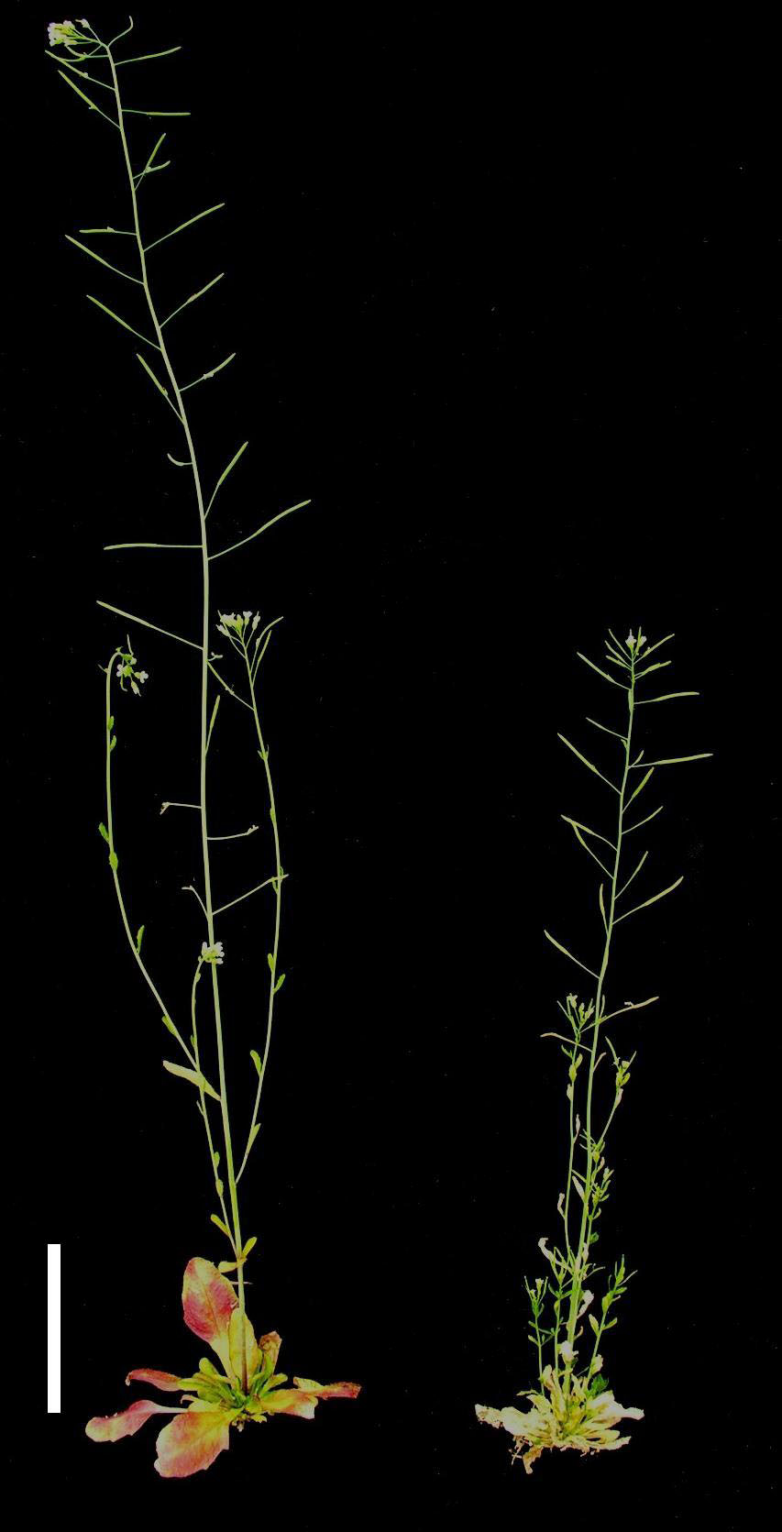
***V. longisporum*-induced stunting and axillary branching in *A. thaliana *ecotype Bur**. Inoculated plant (right) reached 57% of the height of mock-inoculated plant (left) at the onset of fruit maturation and showed bolting of shoots from the axils of the rosette leaves. Bar = 5 cm.

**Figure 6:**
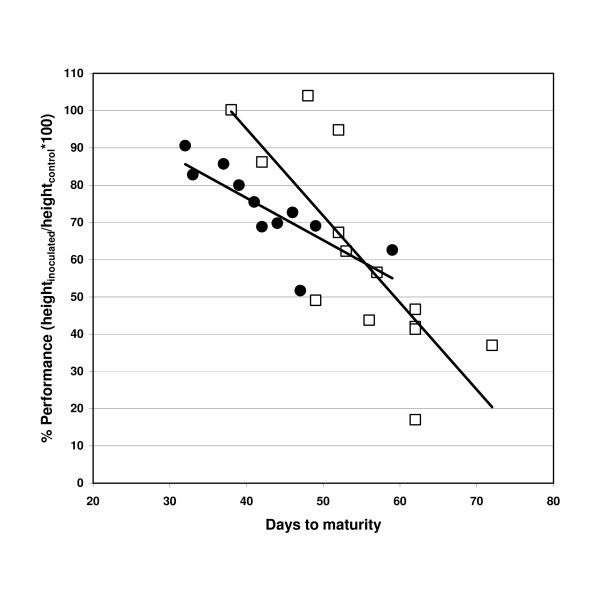
**Correlation of performance with development time in L*er *and Bur**. Performance (%) = Height_inoculated_/Height_control _×100; Ecotypes L*er *(circles) and Bur (squares) are illustrated. Each data point represents a separate infestation experiment. Experiments were performed at different seasons over three years. L*er*: r=-0.79, p = 0.004; Bur: r=-0.79, p < 0.001.

Stunting was a particularly striking disease symptom (Figure 5). Stunting resistance was recorded in two different ways in the present study: 1) As the absolute plant height at the onset of maturity of inoculated plants, and 2) as performance (mean height_inoculated_/mean height_control _×100). The trait performance compensates for the fact that plants carrying a homozygous *erecta *mutation have a much lower plant height than plants carrying a wild type *erecta *allele. The performance of L*er *and Bur varied between different experiments. Bur showed much more variation across different experiments than L*er *(Figure 6). The performance was significantly negatively correlated with the development time that differed between the experiments (Figure 6). While Bur performed better than L*er *when maturity was reached early, it showed much more stunting caused by *V. longisporum *when the development was prolonged due to season. The same effect was observed when the performance of F3-families was correlated with their mean development time: Early-flowering families performed better than late-flowering lines (Table 3). The performance showed a weak positive correlation with colonisation rates in all three F3-experiments (Table 2), meaning that severely colonised lines were less stunted than colonisation-resistant lines. This relationship was even more distinct regarding the absolute plant height of inoculated F3-families (r = 0.62 in E1; *erecta *plants were excluded). As another method to assess stunting, the fresh weight for each plant was recorded. However, this trait varied to a considerably higher degree than plant height due to differences in branching patterns among plants and small-scale variation in nutrient supply and humidity of the soil. In a two-factorial ANOVA, no significant genotype effect on the fresh weight could be detected, whereas the experiment significantly explained fresh weight variance as well as genotype×experiment-interaction (Additional File 5). As expected from traits that are strongly influenced by environmental factors, broad-sense heritability for both plant height and fresh weight was moderate to low (Table 1). No reproducible QTL controlling either the performance or the fresh weight were detected.

As a further approach to identify QTL controlling stunting resistance in the mapping population, we used mean height values of inoculated plants per family after excluding plants with *erecta *phenotype. Significant genotype×experiment-interactions existed for this trait (Additional File 5). In accordance with the strong difference in stunting between seasons, the QTL showed differences in significance level between different infestation experiments. A highly significant locus (*stre1*) could be detected near marker nga111 on chromosome 1. A second significant locus was identified on the same chromosome near marker nga128 (*stre2*). These loci were most significant in the winter experiment E1, but could also be detected in the late spring experiment E3. In E3, three significant QTL controlling the height of *Verticillium*-infected plants (*stre3, stre4 and stre5*) were detected on chromosome 5 (Figure 4, Table 2). *stre3 *and *stre4 *could be reproduced at the putative level in both other experiments and *stre5 *could be reproduced in experiment E1 (Additional file 4). For *stre1 *and *stre2 *on chromosome 1, the alleles increasing the plant height came from L*er*, which is in accordance with the finding that L*er *plants were more resistant to stunting than Bur plants under winter conditions. Whereas *stre1 *showed intermediary inheritance (families from F2 plants heterozygous at marker nga111 show an intermediate phenotype), the *stre2 *allele from L*er *was dominant. For *stre3 *and *4*, plants homozygous for the L*er *alleles were higher than plants that were homozygous for Bur alleles at these loci, but plants that were heterozygous at these loci showed the most extreme stunting phenotype, indicating a complex inheritance of the trait in this region. For *stre5*, the increasing allele came from L*er *and was recessive (Table 2). All QTL for stunting resistance were absent when mock-inoculated plants were analysed, implying these QTL are not general regulators of plant height but selectively conferred stunting resistance under *V. longisporum *pressure. Apart from *erecta*, no reproducible locus controlling plant height in the mock-inoculated plants could be identified with the present data set.

### Two loci on chromosome 1 control *Verticillium*-induced bolting of axillary buds

**Figure 7:**
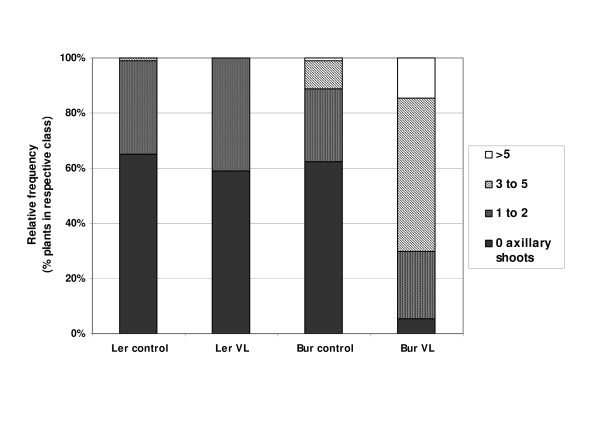
**Proportion of increased axillary branching in L*er *and Bur**. According to their number of axillary rosette shoots plants were assigned to classes ranging from 0 (no axillary rosette shoots) to 3 (more than 5 axillary rosette shoots). No significant difference existed between inoculated (VL) and mock-inoculated (control) L*er *plants (χ^2 ^= 2.42, p = 0.32). Inoculated and mock-inoculated Bur plants differed significantly (χ^2 ^= 93.65, p < 0.0001). N = 90 per column, pooled data of three experiments.

*V. longisporum *increased the bolting of basal axillary buds in Bur. In mock-inoculated plants, typically up to two axillary shoots could be observed, whereas in inoculated plants, often more than five shoots emerged from the axillary buds of the rosette leaves (Figure 5). This phenotype could not be observed in L*er*. In Figure 7 the branching patterns for both parental ecotypes with or without *V. longisporum*-inoculation are compared. The F3-families showed segregation for this trait. The number of plants with a branching score above the overall median was calculated for each F3-family. QTL mapping resulted in two loci on the upper arm of chromosome 1 (*vab1 *and *vab2*) controlling this trait (Figure 4, Table 2). *vab1 *was highly significant in experiment E1, significant in E2 and putative in E3. *vab2 *was significant in E1 and E3, but absent in E2. Alleles increasing axillary branching originated from Bur and were recessive. Accordingly, this phenotype was never observed in F1 plants.

## Discussion

By using a mapping approach it was possible to show that the complex *Verticillium *syndrome in *A. thaliana *is controlled by multiple genes which affect different disease and/or resistance traits separately. Stunting resistance did not depend on resistance to the systemic spread of the pathogen, and both traits were controlled by different QTL. *Verticillium *pathogenesis and disease reactions showed a complex cross-talk with host development and were influenced by environmental factors in greenhouse experiments.

Genetic differences in resistance among natural *A. thaliana*-populations to *Verticillium *systemic spread have not yet been reported in the literature. In a previous study, colonisation rates were determined as colony-forming units obtained by plating macerated tissue of whole plants [29], but no differences between the ecotypes Col-0 and C24 were found before the onset of extensive tissue death. These ecotypes however differed only marginally in their colonisation rates in the shoot apex [44]. The present study shows that the ecotypes Bur and L*er *differ considerably in the degree to which the apical part of the shoot is colonised by *V. longisporum*. The results were reproducible in independent experiments and allowed the detection of QTL controlling the systemic spread of the pathogen. Four *vec *QTL detected in the present study accounted for 91% and 86% of the total trait variation in the experiments E1 and E3, where all four *vec *QTL were detected. The amount of fungal DNA in apical shoot segments correlated significantly with the percentage of colonised shoot segments. The latter reflects the probability of fungal propagules reaching the shoot apex irrespective of the amount of fungal biomass. As expected, the malt agar test was more sensitive in detecting low amounts of colonisation, whereas real-time PCR could differentiate between fungal amounts when shoot colonisation rates were high. Factors that affect the amount of fungal biomass in the shoot apex after systemic colonisation can be determined more reliably by quantitative PCR using genetically homogeneous plant material such as RIL. Furthermore, the distribution of fungal biomass in plant tissue is likely to be inhomogeneous. Increasing the amount of material used for the extraction of DNA for real-time PCR is therefore expected to further reduce the variance of fungal biomass estimates [45]. The strongest QTL controlling systemic spread, *vec1*, explained about 30% of the trait variation and was located on chromosome 2 near the morphological marker *erecta*. There is substantial evidence in the literature that ERECTA itself, a receptor-like kinase with leucine-rich repeats (EC 2.7.11.30), which is mutated in L*er *[37], can play a role in regulating pathogen response in plants [46,47]. In loss-of-function *erecta *mutants callose formation is impaired at the entry sites of the necrotrophic fungus *Plectosphaerella cucumerina *[47], a process that also plays a role in protecting xylem vessels against *Verticillium *infection [48,49]. It is, however, also possible that *vec1 *represents a gene linked to *erecta*. *vec2 *was located near marker nga8 on chromosome 4, in a region where *Vet1 *had been mapped [29]. *Vet1 *was reported to confer resistance to *Verticillium*-induced chlorosis and to act as a negative regulator of flowering. Nothing is known so far about a possible role of *Vet1 *in inhibiting systemic spread. Recent work focusing on genes differentially regulated after infection by *V. longisporum *emphasized the role of apoplastic enzymes. In a microarray experiment a high proportion of genes which encode for apoplastic enzymes were identified among the differentially regulated genes in the *A. thaliana*-*V. longisporum*-pathosystem [50]. Cell wall modifications could be the basis for the inhibition of systemic spread as well as for the stunting phenotype [50]. Up-regulated genes in the regions of the *vec *QTL were At4 g23500, a polygalacturonase (EC 3.2.1.15), and At4 g30460, a glycine-rich protein of unknown function, both localised in the region of *vec3*. Other mechanisms protecting xylem vessels against *V. longisporum *include apoplastic enzymes like β-1.3-glucanases (EC 3.2.1.6), peroxidases (EC 1.11.1.-) and endochitinases (EC 3.2.1.14) as reported for *Brassica *[51]. However, none of the *A. thaliana *genes known to encode cell wall-localised β-1,3-glucanases [52] nor the *A. thaliana *homologue of the chitinase up-regulated through *V. longisporum *in *Brassica *[51] are close to the positions of any *vec *QTL. Vascular occlusions through deposition of phenolic substances in hypocotyl vessels of oilseed rape were found to accompany the resistance phenotype in certain *Brassica *lines [5]. Histological investigations in *A. thaliana *are needed to clarify whether a similar mechanism is responsible for resistance to systemic spread in Bur and if the *vec *QTL could correspond to genes involved in the respective metabolism. Resistance to systemic spread has been distinguished as an important resistance component also in other vascular pathosystems, such as Fusarium head blight of wheat [53]. QTL were detected that specifically control resistance to systemic spread of *Fusarium *[54]. Recently, resistance against northern leaf blight in maize could be dissected into penetration resistance and resistance against the spread of *Setosphaeria turcica *inside the vascular system. Both types of resistance were controlled by different QTL and differed also for their mode-of-action [55]. Dissecting complex disease and resistance phenotypes in a genetic study allows a more in-depth understanding of the genetic and physiological basis of quantitative resistance.

In the present study, resistance to systemic spread in ecotype Bur was associated with slow development, a correlation that became obvious in the F3-families of the (Bur×L*er*) mapping population: Slowly-developing families were less colonised than faster ones. One obvious reason was the overlap of colonisation and developmental QTL. In the present study, the confidence interval of *vec2 *overlapped with those of *dt1 *and *dt2 *(chromosome 4), *vec3 *with *dt3 *(chromosome 4) and *vec4 *with *dt4 *(chromosome 5). Two interpretations for this phenomenon are possible. Either the QTL influencing development have pleiotropic effects on systemic spread or they are linked to QTL controlling systemic spread. Distinguishing between linkage and pleiotropy is important for breeding because only in the case of linkage it would be possible to implement resistance without co-selection of a certain developmental type. Enhanced resistance to systemic spread in slowly-developing genotypes was also observed for certain accessions of *B. oleracea *and *B. rapa *(E. Diederichsen, unpublished results). The localisation of the *vec *and *dt *QTL in the present study indicates that the correlation between both traits is due to linkage rather than pleiotropic effects of the same gene(s). Candidate genes located in the region of *dt1 *are *Vet1 *[29], and *fri*, which is known to be impaired in L*er *[56]. Further studies are required to determine whether *dt1*, *vec2 *and *Vet1 *are identical. Flowering genes in the region of *dt2 *include *cry1*/*hy4*, a blue-light receptor [57], *det1*, a suppressor of photomorphogenesis [58] and *ted1*, an antagonist of *det1 *[59]. A known flowering gene in the region of *dt3 *is *fca *at 9.2 Mb [60,61], which promotes the transition to flowering in the autonomous pathway. *dt4 *possibly represents two dominant genes delaying development with additive effect originating from different parents. This would explain why heterozygotes at marker position nga151 developed significantly more slowly than either maternal or paternal homozygotes. A flowering QTL near marker nga151 was also detected in two different RIL populations [34]. *constans *(*co*), *fy *and *flowering locus C *(*flc*) are possible candidates for this QTL. FLC is a potent inhibitor of the transition to flowering integrating the autonomous and the vernalisation pathway [62]. Bur most likely carries a loss-of-function *flc *allele [63], whilst L*er *has a weak *flc *allele [64]. The detected epistatic interaction between EH4-1, which is close to *fri*, and nga151 in the vicinity of *flc *may suggest that the pathway nevertheless plays a role in the present mapping population. However, a *fri*-independent late-flowering QTL from Bur on chromosome 5 was described as well which could correspond to *dt4 *[63].

Contrary to expectations, high colonisation rates did not coincide with severe disease symptoms such as stunting. Actually a weak positive correlation between colonisation and performance was observed in all three infestation experiments, showing that heavily colonised families were less stunted than sparsely colonised ones. This correlation does not necessarily reflect a causal relationship. Both systemic spread and performance were negatively correlated with development time, a factor that might influence both traits independently. The weaker performance of slowly-developing plants seems to have a physiological basis: In both parental lines Bur and L*er*, performance decreased when development was slowed down as a consequence of seasonal changes. An unknown regulatory mechanism might control both stunting severity and developmental velocity differently in interaction with seasonal influences still effective in the greenhouse. Temperature, seasonal changes in light intensity and a higher dosage of blue light in summer are potential factors causing summer/winter differences. Nevertheless, host genetic disposition also plays a role, since Bur shows a much higher difference in performance between summer and winter. In summer experiments with short development times, Bur was consistently less stunted than L*er*, whereas the opposite was true in winter experiments. The dependence of quantitative resistance on developmental aspects is a frequently recognised phenomenon [18,19,65,66]. Seasonal differences could also be observed in significance levels of stunting resistance QTL. In E1 (started in January 2007), *stre1 *and *stre2 *were strong, in E3 (started in April 2008) they were present, but only at the putative level. *stre3 *to *stre5 *were strong in E3, while in E1 and E2 they were weak or absent (Additional File 4). The absence of most stunting resistance QTL in E2 (started in March 2007) might have been due to inhomogeneous soil used in this experiment so that nutritional effects masked genetic effects or seasonal impact on development. The finding that the resistant alleles of the *stre *QTL on chromosome 1 were of L*er *origin is in good accordance with L*er *having been more resistant to stunting in winter. However, the situation for *stre3 *to *stre5 *is more complex. Only for *stre5*, the resistance allele came from L*er*. For *stre3 *and *4*, heterozygous families were more susceptible than families carrying either of the parental alleles in the homozygous state. A possible explanation would be that *stre3 *and *stre4 *contain an array of susceptibility genes of different origin with additive effects. A gene recently found to be involved in *Verticillium *resistance is *rfo1*, a receptor-like kinase (EC 2.7.11.30) otherwise mediating resistance to *Fusarium oxysporum *[26]. The *rfo1*-allele from the *A. thaliana *accession 'Taynuilt' (Ty-0) conferred significant resistance against fresh weight loss caused by *Verticillium *and is located at the bottom of chromosome 1 at 29.9 Mb. Thus it could be a candidate for stunting resistance QTL *stre1 *near marker nga111 at 27.3 Mb, but stunting resistance was not observed for L*er *in the respective study [26]. The results of the present study suggest that the symptoms stunting and systemic spread are controlled by different pathways. This underlines the observation that shoot colonisation is not a prerequisite to cause damage to the host in greenhouse or growth chamber assays, which has also been described before for *Brassica *[2,51]. As stunting can occur without detectable amounts of *V. longisporum *in the shoot, a translocated signal is likely to be involved in the induction of stunting. *Verticillium *has been shown to interfere with various signalling pathways, like ABA, ethylene, jasmonic acid and salicylic acid [26,27,29,67]. Some components are obviously important for conferring resistance, since mutants in the ethylene or ABA signalling components *ein2*, *ein4*, *ein6 *and *aba2 *are more susceptible to *Verticillium *than wild type in terms of chlorosis and/or stunting. However, some regulatory mechanisms, especially ethylene production, also seem to be manipulated in a way that enhances disease symptoms in the host plant [27,68]. Plants deficient for the ethylene receptor *etr1 *showed enhanced resistance in terms of chlorosis [27,29] and fresh weight loss [26] compared to wild type. Our understanding of the nature and exact role of fungal and plant signals in the context of this pathosystem is only at the beginning [69]. A fungal elicitor protein, *Verticillium dahliae*-necrosis-and-ethylene-inducing factor (VdNEP), was found to be involved in symptom development in cotton [70] and is also present in *V. longisporum *(Weiberg and Karlovsky, unpublished results), emphasizing the role of ethylene induction in *Verticillium *pathogenesis.

The alteration of the *A. thaliana *branching pattern after *Verticillium *infection has previously been observed for the *A. thaliana *ecotypes C24 and 'Coimbra' (Co-1) [29]. Scoring the (Bur×Ler) F3-families for basal branch numbers gave clear results for all three experiments. Both loci were on chromosome 1 and together accounted for 72% of the total variation in experiment E1. *vab1 *had a stronger effect and was located shortly below marker F16J7-TRB (3.8 Mb, AGI physical map). A promising candidate for *vab1 *could be *supershoot *(*sps*) at 5.6 Mb. It encodes a P450 cytochrome oxidase (EC 1.9.3.1) that regulates axillary branching by locally modulating cytokinin levels in the leaf axils [71]. Allelic variation of *sps *is significantly associated with basal branching patterns among natural *A. thaliana *populations [72]. A possible explanation for the differences in the present study would be that *Verticillium *interferes with cytokinin or other hormonal regulatory pathways, but not every natural *sps *allele responds to phytohormones in the same way. Other possible candidates for *vab1 *include *bud1 *at 6.3 Mb, encoding a MAP kinase kinase (EC 2.7.12.2) involved in auxin transport and systemic acquired resistance [73], and *mp*, an auxin-response factor at 6.9 Mb [74]. *vab2*, the weaker branching QTL, was located between markers nga392 at 9.8 Mb and EH1-2 at 12.8 Mb. A gene in this region influencing branching pattern is *axr*3 at 11.6 Mb involved in auxin signalling [75]. Whereas ethylene and ABA-signalling have been in the focus of *Verticillium *researchers [26,27,29], there is no evidence so far for a possible role of cytokinin and auxin. The findings of the present study show that the roles of cytokinin and auxin signalling in *Verticillium *pathogenesis require further investigation.

The search for genetic resources mediating *Verticillium *resistance in *Brassica *has been difficult because qualitative resistance relying on a single gene is not available for this pathosystem. In breeding, quantitative resistance is more difficult to implement as it is mostly controlled by several genes with small effects, and it demands more effort to introgress quantitative resistance. Nevertheless, it is of high interest to breeders since it is often more durable than qualitative resistance. For some pathogens it is the only resistance available [12,18,19]. In greenhouse experiments, *A. thaliana *has proven to be a good model for genetic studies on quantitative resistance against *V. longisporum *in crucifers. The complexity of *Verticillium *resistance in *A. thaliana *was particularly striking for stunting resistance, which was highly conditional on environmental factors in the present study. A combination of several QTL was necessary to confer good growth performance regardless of different developmental patterns and growing conditions. Fungal spread within the plant was not a prerequisite for increased symptom severity in *A. thaliana*, but is likely to play a greater role for *Brassica *crops in the field, as host colonisation enables the fungus to use more host resources for its own proliferation. In *Brassica*, disease symptoms were indeed correlated with the presence of *V. longisporum *in shoots [[5,24], S. Konietzki, FU Berlin, personal communication]. Breeding for resistance to systemic spread should be a promising strategy to control this disease in *Brassica*. The present genetic study in *A. thaliana *provides the basis for the identification of individual resistance genes, their cloning, and the elucidation of the resistance mechanisms involved.

## Conclusions

*A. thaliana *can be used as a model to study typical features of the *V. longisporum *pathosystem, such as host genotype and developmental effects on pathogenesis and resistance components. Systemic spread of *V. longisporum *in the host as well as resistance to this major disease trait are described for the first time in natural *A. thaliana *accessions. This creates the possibility to study a major resistance mechanism against vascular pathogens in this model plant and to clone relevant genes of the involved pathways using the Arabidopsis tool box. Stunting resistance and resistance to systemic spread were controlled by different QTL and should be treated as separate traits. Developmental and environmental effects on pathogenesis and resistance need to be considered when designing and interpreting experiments in research and breeding. Further studies will help to determine the exact role of potential candidate genes.

## Authors' contributions

EH planned and carried out all plant experiments, all experiments related to mapping such as marker analysis, mapping and QTL analysis and drafted the manuscript. ED conceived of the study, participated in its design and coordination and helped to draft the manuscript. PK provided the qPCR data on fungal biomass and helped to draft the manuscript. All authors read and approved the final manuscript.

## Supplementary Material

Additional file 1 **New sequence-characterised (SCAR) markers developed for (Bur×L*er*) mapping populations**. New markers are listed with name, chromosomal position according to the AGI map, sequences of forward and reverse primers, the annealing temperature for PCR-amplification and the fragment size for Bur and L*er *respectively. Length polymorphisms were identified using the MSQT query tool [36]. Click here for file

Additional file 2 **Linkage map for the (Bur×L*er*) F2 mapping population**. The linkage map shows the five *A. thaliana *chromosomes containing all markers that were analysed. Physical positions of markers according to the AGI map and marker distances in cM as determined in the F2 population are displayed. Click here for file

Additional file 3 **Frequency distributions for trait values of F3-families in individual infestation experiments**. Frequency distributions of F3-family values are shown for all three infestation experiments for the following traits: degree of *Verticillium *colonisation, development time, stunting resistance and *Verticillium*-induced axillary branching. Parental and F1-values are indicated by boxes. Click here for file

Additional file 4 **QTL information for individual infestation experiments**. Peak positions, LRS values and significance levels of QTL in the individual infestation experiments are listed for the following traits: degree of *Verticillium *colonisation, development time, stunting resistance and *Verticillium*-induced axillary branching. Click here for file

Additional file 5 **Tests of between-subjects effects in two-factor ANOVA**. Two-factor ANOVA was performed to test the influence of genotype and experiment and their interaction on trait values. Results of tests of between-subjects effects are shown for the traits development time, fresh weight of inoculated plants, and the height of inoculated plants (height of plants with and without *erecta *phenotype were tested separately). Click here for file
