# Supplementary material for: Genetic and environmental control of the Verticillium syndrome in Arabidopsis thaliana
Source: BMC Plant Biol. 2010 Nov 2;10:235. doi: 10.1186/1471-2229-10-235 (PMC3017855; doi:10.1186/1471-2229-10-235)
Supplement: Additional file 1 — New sequence-characterised (SCAR) markers developed for (Bur×Ler) mapping populations. New markers are listed with name, chromosomal position according to the AGI map, sequences of forward and reverse primers, the annealing temperature for PCR-amplification and the fragment size for Bur and Ler respectively. Length polymorphisms were identified using the MSQT query tool [36]. [file 1471-2229-10-235-S1.PDF]

Additional file 1 – New sequence-characterised markers developed for (Bur×L*er*) mapping populations

The physical positions refer to the map of the Arabidopsis Genome Initiative (AGI). Chr. = chromosome, T_a=annealing temperature, size of fragments in base pairs (bp).

Marker name	Chr	Physical position (bp)	Forward primer	Reverse primer	T_a	Size L*er*	Size Bur
EH1-1	1	2696950	AGTTGGGTATAGGAAAAAGCTT	CCTCCGATTTCACGATCAACCGC	55	195	151
EH1-2	1	12892640	GCGAGCTTCCTAAGTAAGAC	CGCTGGGTGAGGTATGTCG	55	127	139
EH1-3	1	22096879	CGACGACGTATAGGCTTTGT	CAGAGAGGAAGAGCTAAACGG	55	238	211
EH2-1	2	2365239	GGCAGCAACTTACAATAAATGGAGG	GAGTGGCTGCTATTTGGCC	55	193	160
EH2-3a	2	6280304	AAGATTGTGAAAGTAGTGATGAT	ATGTTCGACAAAATGCACCA	50	158	194
EH2-4	2	10050145	CACCACGAAAGTGACACTAC	CGGTCAAAACAACGTGGTC	55	250	283
EH3-1	3	7842375	CTGTGGTGTTAGAGCTACAGG	CCAACCCGGTCACAAACCTGC	55	313	352
EH3-3	3	10849502	GACTTTGTAGAATGAGTAGTCAA	CATGATCATAACCATCGTCTGG	55	199	189
EH4-1	4	2440967	CGCAGCGTCTTCCTTGTAAATCTTG	GTCAATAGAGTCTCACAGATGCG	55	260	285
EH5-7	5	16565648	GAGTTCCTCTTGGTTGTTGG	GTGTATCGGTAGAAACAAGCG	55	163	134
EH5-9	5	18988084	CACAGGTACAGGTTCTTCAGGC	GGTTTACTTCTGAATCTCCG	55	300	312
