# Supplementary material for: Genetic and environmental control of the Verticillium syndrome in Arabidopsis thaliana
Source: BMC Plant Biol. 2010 Nov 2;10:235. doi: 10.1186/1471-2229-10-235 (PMC3017855; doi:10.1186/1471-2229-10-235)

Additional File 2

Linkage map for *Arabidopsis thaliana* (BurxL*er*) mapping populations

Marker names are listed on the right side of each chromosome together with their physical positions on the map of the Arabidopsis Genome Initiative (AGI) in base pairs (bp) if known. Genetic distances (in cM) are shown on the left side of each chromosome.

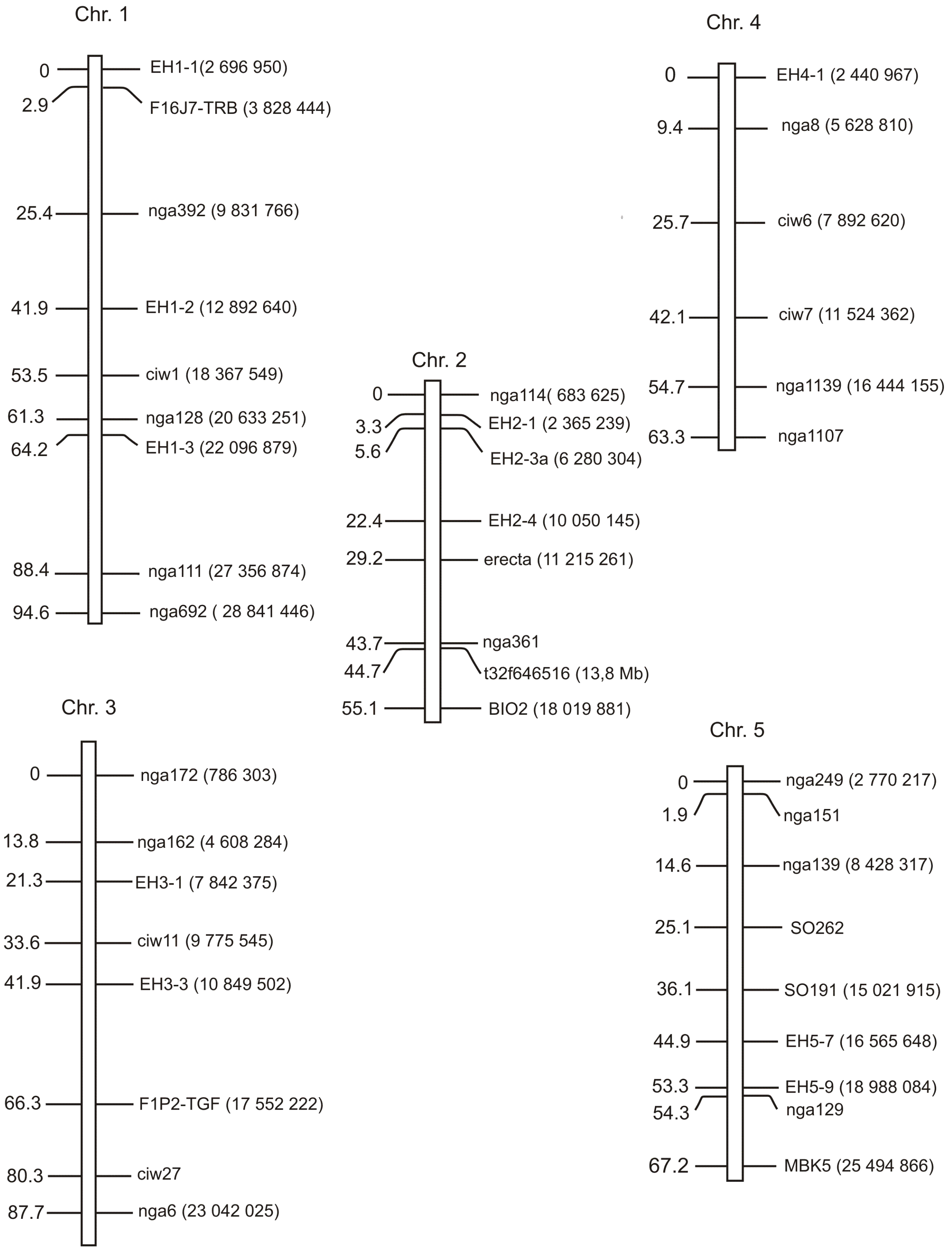

Supplement: Additional file 2 — Linkage map for the (Bur×Ler) F2 mapping population. The linkage map shows the five A. thaliana chromosomes containing all markers that were analysed. Physical positions of markers according to the AGI map and marker distances in cM as determined in the F2 population are displayed. [file 1471-2229-10-235-S2.PDF]
