# Supplementary material for: Genetic and environmental control of the Verticillium syndrome in Arabidopsis thaliana
Source: BMC Plant Biol. 2010 Nov 2;10:235. doi: 10.1186/1471-2229-10-235 (PMC3017855; doi:10.1186/1471-2229-10-235)

Additional File 3: Frequency distributions for trait values of F3-families in individual infestation experiments.

The values for parental and F1-generations are indicated by boxes.

Frequency distributions of trait "*Verticillium* systemic colonisation"
(% colonised shoot segments/total shoot number of inoculated plants)

Experiment 1

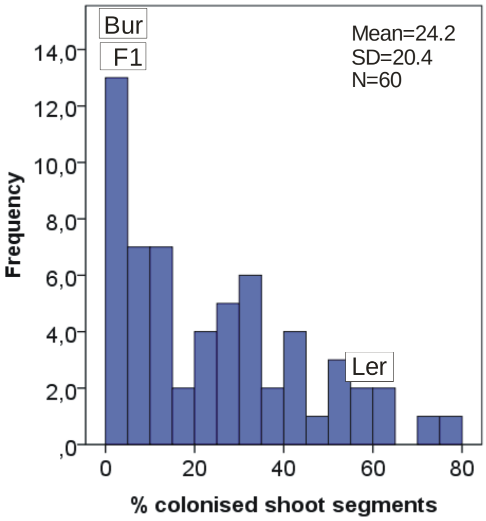

Experiment 2

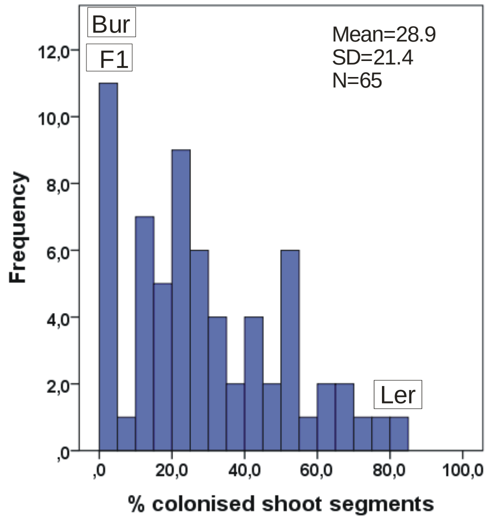

Experiment 3

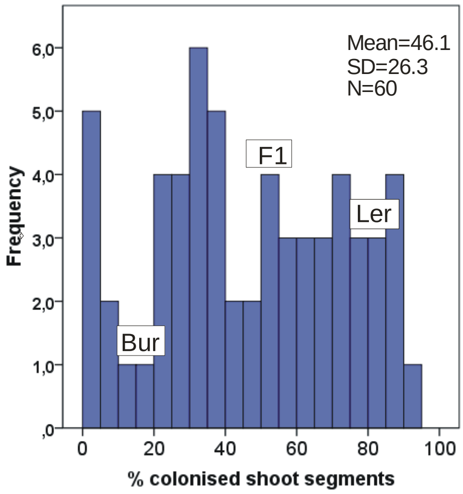

Frequency distributions of trait "development time"
(days from germination to onset of maturity in mock-inoculated plants
F3-family means)

Experiment 1

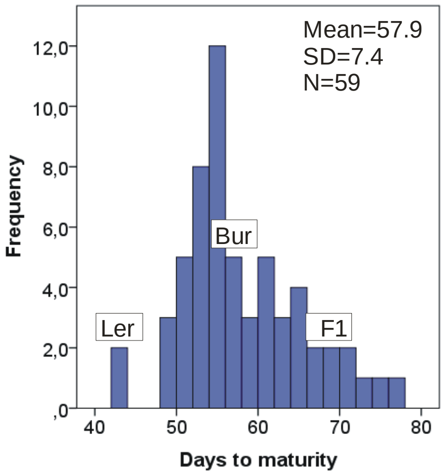

Experiment 2

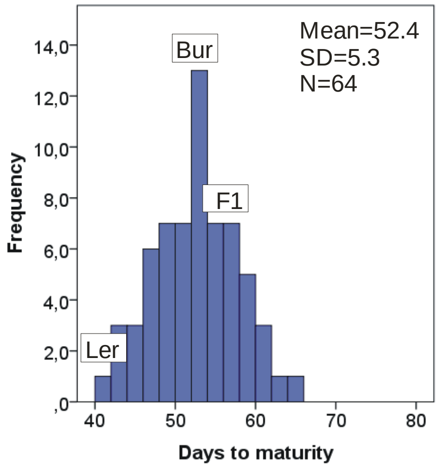

Experiment 3

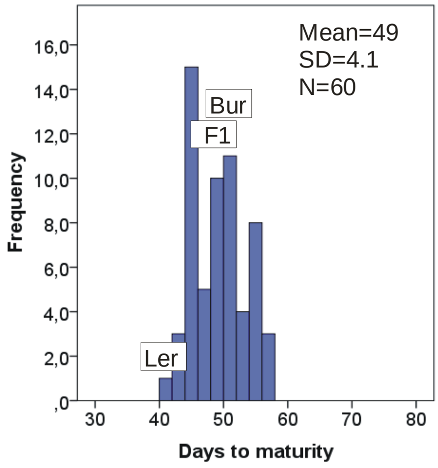

Frequency distribution of trait "stunting resistance" (F3-family means of height of inoculated plants; plants with *erecta* phenotype excluded)

Experiment 1

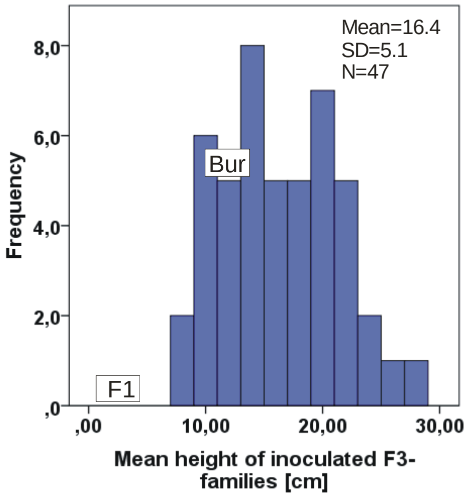

Experiment 2

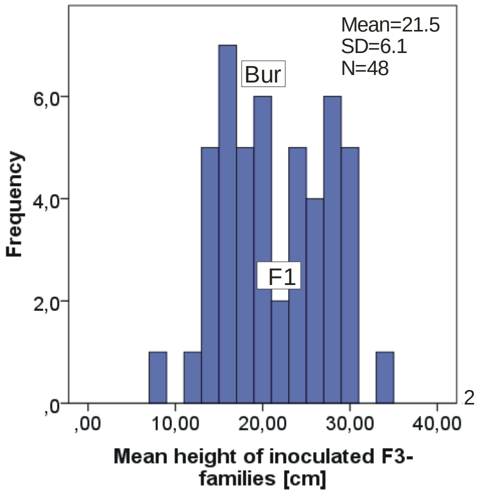

Experiment 3

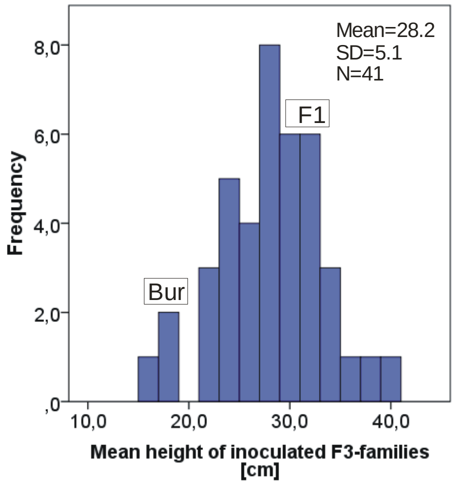

Frequency distributions of trait '*Verticillium*-induced axillary branching' (number of inoculated plants with branching score above median per F3-family)

Experiment 1

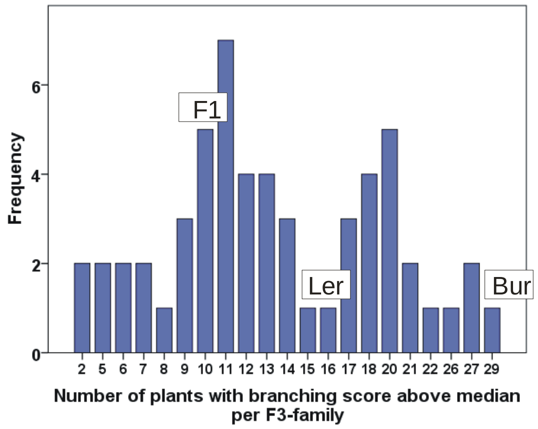

Experiment 2

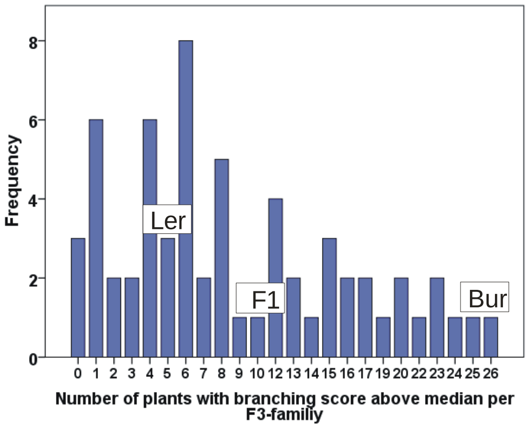

Experiment 3

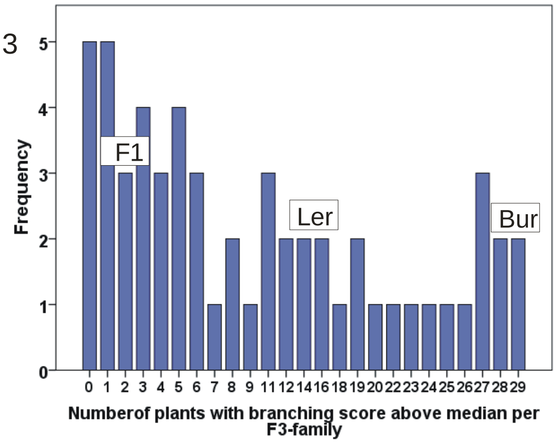

Supplement: Additional file 3 — Frequency distributions for trait values of F3-families in individual infestation experiments. Frequency distributions of F3-family values are shown for all three infestation experiments for the following traits: degree of Verticillium colonisation, development time, stunting resistance and Verticillium-induced axillary branching. Parental and F1-values are indicated by boxes. [file 1471-2229-10-235-S3.PDF]
