# Supplementary material for: Genetic and environmental control of the Verticillium syndrome in Arabidopsis thaliana
Source: BMC Plant Biol. 2010 Nov 2;10:235. doi: 10.1186/1471-2229-10-235 (PMC3017855; doi:10.1186/1471-2229-10-235)
Supplement: Additional file 4 — QTL information for individual infestation experiments. Peak positions, LRS values and significance levels of QTL in the individual infestation experiments are listed for the following traits: degree of Verticillium colonisation, development time, stunting resistance and Verticillium-induced axillary branching. [file 1471-2229-10-235-S4.PDF]

Additional file 4 – QTL information for individual infestation experiments

LRS= likelihood ratio statistic. Asterisks denote genome-wide significance thresholds determined by permutation tests as follows: * 37%, ** 95%, ***99.9%

Trait	QTL	Chr	Peak Pos. (cM)	Experiment	LRS
Degree of *Verticillium* colonisation (*vec*; malt agar test; number of colonised shoot segments/total number of shoot segments in %)	*vec1*	2	26	E1	21.2**
			29	E2	6.6$^{n.s.}$
			27	E3	20.5**
	vec2	4	9	E1	8.6*
			10	E2	9.4*
			16	E3	10.0*
	vec3	4	42	E1	9.0*
			-	E2	n.s.
			45	E3	17.0**
	vec4	5	11	E1	20.7**
			15	E2	7.6*
			20	E3	8.3*
Development time (*dt*; days from germination to maturity in mock-inoculated plants)	*dt1*	4	5	E1	18.8**
			3	E2	20.3**
			5	E3	20.6**
	dt2	4	16	E1	18.2**
			-	E2	n.s.
			10	E3	20.6**
	dt3	4	33	E1	17.2**
			-	E2	n.s.
			31	E3	15.8**
	dt4	5	5	E1	13.3*
			5	E2	17.0**
			4	E3	13.3*
Stunting resistance (*stre*; shoot height of *Verticillium*-inoculated plants in cm)	*stre1*	1	79	E1	32.5***
			-	E2	n.s.
			80	E3	11.6*
	stre2	1	59	E1	22.2**
			-	E2	n.s.
			59	E3	10.8*
	stre3	5	32	E1	7.5*
			27	E2	9.2*
			31	E3	20.4**
	stre4	5	38	E1	7.6*
			39	E2	8.1*
			39	E3	20.9**
	stre5	5	60	E1	12.2*
			-	E2	n.s.
			53	E3	15.8**
Verticillium-induced axillary branching (*vab*; number of plants with a branching score above the overall median of *Verticillium*-inoculated plants)	*vab1*	1	9	E1	28.1***
			16	E2	20.8**
			19	E3	12.2*
	vab2	1	34	E1	22.2**
			-	E2	n.s.
			32	E3	17.3**
