# Supplementary material for: Genetic and environmental control of the Verticillium syndrome in Arabidopsis thaliana
Source: BMC Plant Biol. 2010 Nov 2;10:235. doi: 10.1186/1471-2229-10-235 (PMC3017855; doi:10.1186/1471-2229-10-235)
Supplement: Additional file 5 — Tests of between-subjects effects in two-factor ANOVA. Two-factor ANOVA was performed to test the influence of genotype and experiment and their interaction on trait values. Results of tests of between-subjects effects are shown for the traits development time, fresh weight of inoculated plants, and the height of inoculated plants (height of plants with and without erecta phenotype were tested separately). [file 1471-2229-10-235-S5.PDF]

Additional File 5: Tests of between-subjects effects in two-factor ANOVA

Two-factor analysis of variance was performed to assess the effects of genotype, experiment and their interaction. The analysis is based on the data from genotypes overlapping between infestation experiments E1 and E3. For parameter "Plant height", analyses were performed for plants with and without *erecta* phenotype separately.

Dependent variable: Development time (days from germination to maturity of first siliques in mock-inoculated plants)

Source		Type III square sum	df	Mean square	F	Sig.
Constant term	Hypothesis	3131553,189	1	3131553,189	151,786	0,042
	Error	22518,767	1,091	20631,331		
Experiment	Hypothesis	19746,999	1	19746,999	119,035	0,000
	Error	6039,974	36,409	165,892		
Genotype	Hypothesis	39281,808	36	1091,161	6,361	0,000
	Error	6175,442	36	171,540		
Experiment * genotype	Hypothesis	6175,442	36	171,540	7,043	0,000
	Error	26645,783	1094	24,356		

Dependent variable: Fresh weight of inoculated plants

Source		Type III square sum	df	Mean square	F	Sig.
Constant term	Hypothesis	425,246	1	425,246	4,206	0,289
	Error	100,941	,998	101,116		
Experiment	Hypothesis	101,203	1	101,203	86,350	0,000
	Error	42,194	36,001	1,172		
Genotype	Hypothesis	39,074	36	1,085	,926	0,591
	Error	42,206	36	1,172		
Experiment * genotype	Hypothesis	42,206	36	1,172	23,003	0,000
	Error	108,714	2133	,051		

Dependent variable: Height of inoculated plants without *erecta* phenotype

Source		Type III square sum	df	Mean square	F	Sig.
Constant term	Hypothesis	405194,509	1	405194,509	12,193	0,172
	Error	34136,109	1,027	33233,051		
Experiment	Hypothesis	32447,542	1	32447,542	132,710	0,000
	Errror	6965,939	28,491	244,500		
Genotype	Hypothesis	26106,092	27	966,892	2,913	0,004
	Error	8613,716	25,949	331,949		
Experiment * genotype	Hypothesis	8564,765	26	329,414	8,764	0,000
	Error	44277,362	1178	37,587		

Dependent variable: Height of inoculated plants with *erecta* phenotype

Source		Type III square sum	df	Mean square	F	Sig.
Constant term	Hypothesis	38373,470	1	38373,470	10,914	0,179
	Error	3659,679	1,041	3516,106		
Experiment	Hypothesis	3653,656	1	3653,656	102,882	0,000
	Error	1446,811	40,740	35,513		
Genotype	Hypothesis	6075,519	30	202,517	3,648	0,001
	Error	1461,860	26,330	55,520		
Experiment * genotype	Hypothesis	1447,361	27	53,606	3,729	0,000
	Error	10394,512	723	14,377		
